# Access to the CNS: Biomarker Strategies for Dopaminergic Treatments

**DOI:** 10.1007/s11095-017-2333-x

**Published:** 2018-02-15

**Authors:** Willem Johan van den Brink, Semra Palic, Isabelle Köhler, Elizabeth Cunera Maria de Lange

**Affiliations:** Division of Systems Biomedicine and Pharmacology, Leiden Academic Centre for Drug Research, Leiden University, Einsteinweg 55, 2333 CC Leiden, The Netherlands

**Keywords:** biomarkers, CNS drug development, dopaminergic agents, systems pharmacology

## Abstract

Despite substantial research carried out over the last decades, it remains difficult to understand the wide range of pharmacological effects of dopaminergic agents. The dopaminergic system is involved in several neurological disorders, such as Parkinson’s disease and schizophrenia. This complex system features multiple pathways implicated in emotion and cognition, psychomotor functions and endocrine control through activation of G protein-coupled dopamine receptors. This review focuses on the system-wide effects of dopaminergic agents on the multiple biochemical and endocrine pathways, in particular the biomarkers (i.e., indicators of a pharmacological process) that reflect these effects. Dopaminergic treatments developed over the last decades were found to be associated with numerous biochemical pathways in the brain, including the norepinephrine and the kynurenine pathway. Additionally, they have shown to affect peripheral systems, for example the hypothalamus-pituitary-adrenal (HPA) axis. Dopaminergic agents thus have a complex and broad pharmacological profile, rendering drug development challenging. Considering the complex system-wide pharmacological profile of dopaminergic agents, this review underlines the needs for systems pharmacology studies that include: i) proteomics and metabolomics analysis; ii) longitudinal data evaluation and mathematical modeling; iii) pharmacokinetics-based interpretation of drug effects; iv) simultaneous biomarker evaluation in the brain, the cerebrospinal fluid (CSF) and plasma; and v) specific attention to condition-dependent (e.g., disease) pharmacology. Such approach is considered essential to increase our understanding of central nervous system (CNS) drug effects and substantially improve CNS drug development.

## Introduction

Over the last decades, the development of therapies targeting diseases affecting the central nervous system (CNS) has been facing numerous challenges while the number of people suffering from CNS disorders has tremendously grown, exceeding one billion worldwide nowadays (1,2). The challenges mostly rely on the insufficient knowledge of biomolecular mechanisms underlying many CNS-related diseases, as well as the poor understanding of mechanisms of action of many CNS drugs. In order to improve drug efficacy, both pharmaceutical industry and academic community have fostered the implementation of biomarker-based approaches for translational pharmacology and dose decision-making in clinical settings. A biological or biochemical marker represents a measurable sign with regard to a pharmacological or pathological process, providing a clinically meaningful endpoint in predicting the effect of a chosen treatment (3–5). Biological markers are recognized as a valuable tool in drug development, allowing for further elucidation of both drug efficacy and side effects. CNS drug discovery and development faces multiple challenges, including the large number of drugs that fail in late phases of clinical trials due to poor understanding of processes underlying the dose response relation (6). In this context, biomarkers represent an attractive alternative approach to support identification of most promising compounds, guide the dosing strategies in early clinical trials, and help recognizing a patient population that is most likely to benefit from a specific treatment.

This systematic and exhaustive review presents all biochemical indicators that have been previously reported as being related to dopaminergic drug effects, as well as their potential role in biomarker-driven CNS drug development, focusing on biomarkers in rodents biofluids, specifically brain extracellular fluid (brain_ECF_), cerebrospinal fluid (CSF), plasma and urine.

### Anatomy and Physiology of the Dopaminergic System

**Fig. 1:**
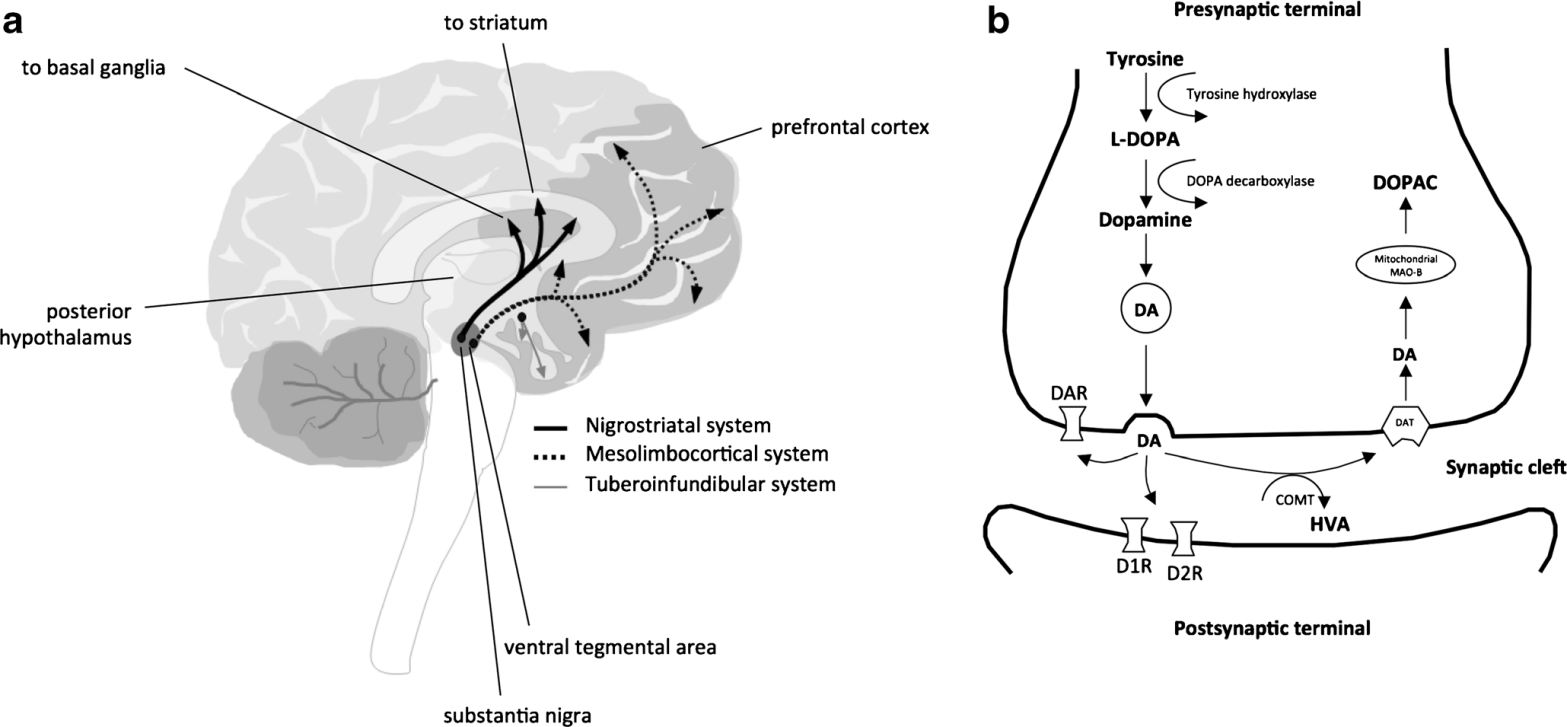
Overview of the dopaminergic system. **A** Representation of the dopamine pathway architecture in the brain. **B** Illustration of the dopamine production and degradation, as well as the synaptic signaling.

Dopamine is a neurotransmitter that belongs to the catecholamine family and is primarily synthesized in the brain and the kidneys. In the brain, dopamine is produced in the cell bodies of dopaminergic neurons located in the substantia nigra (SN), the ventral tegmental area (VTA) and the hypothalamus. These neurons send projections to multiple brain areas where dopamine is stored and released, including the striatum (nigrostriatal pathway), the prefrontal cortex (PFC) (mesocortical pathway), the nucleus accumbens (NAc) (mesolimbic pathway) and the pituitary gland (tuberoinfundibular pathway), as illustrated in Fig. 1. It should be noted that these pathways do not represent all dopamine systems in the brain. Other systems, such as the thalamic dopamine system, are increasingly recognized as important additional components of the brain dopamine pathways (7). The presence of dopamine in the mesolimbic pathway is related to positive reinforcement, reward and/or pleasure, while in mesocortical pathway it is involved in cognitive control of behavior. Furthermore, the role of dopamine in the nigrostriatal pathway, transmitted from the SN (midbrain) to the putamen in the dorsal striatum, is to simulate reward-related cognitive processes as well as psychomotor function. The tuberoinfundibular pathway projects dopaminergic neurons from the hypothalamus to the pituitary gland to modulate secretion of hormones, including prolactin. Dopaminergic pathways also project from the VTA (midbrain) to the amygdala, the hippocampus, and the cingulate cortex. As such, dopamine is simultaneously involved in both emotional and memory processing. Dopaminergic neurons form a tight network with a number of other neuronal pathways, including choline, glutamate and gamma-aminobutyric acid (GABA) systems, showing its possible role in multiple complex processes. Therefore, any drug targeting the dopaminergic neurons may influence multiple transduction pathways including both the dopaminergic and other systems.

Five dopamine receptor subtypes, often referred to as D_1–5_ receptors, have been reported in the CNS, all being G-protein coupled receptors that may function independently but of which the downstream pathways may also interact (8). Dopamine receptors are divided into D_1_- and D_2_-like receptor classes, the D_1_ receptor class including D_1_ and D_5_ receptors while D_2_ receptor class includes D_2_, D_3_, and D_4_ receptors. D_1_ receptor and D_2_ receptor classes have opposing effects with regard to adenylyl cyclase activity, cAMP concentrations, as well as phosphorylation of proteins, resulting in either stimulatory or inhibitory action on voltage-gated and ion channels in synapses (9). D_1_ receptor are highly expressed in the striatum, NAc, SN, frontal cortex and amygdala, while lower expression of D_1_ receptor is found in the hippocampus, thalamus, and cerebellum. D_2_ receptor are mainly localized in the striatum, NAc, SN, hypothalamus, cortical areas, amygdala and hippocampus. Although dopamine receptors are most densely expressed in the brain, they are also found in the periphery in different patterns of expression (10), highlighting the system-wide effects of dopamine that are crucial in maintaining homeostasis.

### Dopaminergic Agents for Treatment of Neurological Disorders

The dopaminergic system has been exploited for treatment opportunities in a large variety of disorders. Due to its broad implication in pathophysiology, the current pharmacological efforts mostly focus on targeting both the dopamine receptors and subsequent post-receptor mechanisms. Different types of dopaminergic drugs have been developed so far, primarily dopamine agonists and dopamine antagonists.

#### Dopamine Agonists

Dopamine agonists have been developed for treating Parkinson’s disease, a progressive neurodegenerative disorder presenting both motor and non-motor symptoms. The pathology of the Parkinson’s disease is characterized with an extensive loss of dopamine neurons in the SN and accumulation of the protein α-synuclein in Lewy bodies within nerve cells in specific brain regions (11). Although the underlying mechanisms leading to Parkinson’s disease remain poorly understood, a strong association between low dopamine brain levels and Parkinson’s disease symptoms has been frequently reported (12). Dopamine receptor agonists, introduced first in 1970 for the treatment of Parkinson’s disease, act directly on dopamine receptors to mimic endogenous neurotransmission. Levodopa (L-DOPA), a pro-drug crossing the blood-brain barrier (BBB), was the first therapeutic option available for treating Parkinson’s disease. Various other agonists, e.g., apomorphine, bromocriptine and pramipexole, have been later developed and commercialized, showing comparable effectiveness (13).

#### Dopamine Antagonists

While most of the currently available dopamine agonists are used for Parkinson’s disease, the vast majority of dopamine antagonists have been developed for the treatment of schizophrenia. Multiple studies using animal models of schizophrenia have elucidated a pattern of persistent hyperdopaminergic state, accompanied with altered stimulus recruits of dopamine in different brain regions. Cognitive impairments during psychosis might thus be explained by a rapid release of dopamine into the mesolimbic and the nigrostriatal regions (14). Chlorpromazine was the first and extremely potent antagonist of D_2_ receptor discovered, which considerably fostered antipsychotic drug development. Nevertheless, chlorpromazine treatment is accompanied with pronounced adverse effects, including neuroleptic malignant syndrome and extrapyramidal symptoms (EPS) such as tardive dyskinesia. Other D_2_ receptor antagonists, e.g., haloperidol, risperidone and clozapine, have been developed to exhibit comparable or greater effectiveness with fewer of these side effects, in particular EPS (15,16).

Many of dopaminergic agents were discovered with incomplete understanding of their modes of action, often resulting in unpredictable side effects and/or off-target effects. It is only after having been introduced to market that studies were conducted to elucidate their modes of actions, which revealed multiple pathways affected (17–19).

#### Selectivity of Dopaminergic Drugs

Clozapine is currently the “gold standard” for the treatment of schizophrenia(15). Interestingly, this is one of the least selective D_2_ receptor antagonists (16,20). Indeed, schizophrenia is a polygenic disease, and therefore a ‘shotgun-approach’ may be more successful than a ‘magic-bullet approach’ (16). Many D_2_ receptor antagonists have therefore affinity for more receptors, including serotoninergic, adrenergic, muscarinic, and histaminergic receptors (16,20). Also many D_2_ receptor agonists were found non-selective, with affinity for other dopaminergic, serotonergic, adrenergic and histaminergic receptors (21). This should be taken into consideration when evaluating the effects of these agents on the system-wide biochemical pathways.

This review aims to further improve the understanding of mechanisms of action by providing an extensive overview of the pathways that are affected by dopaminergic agents, with the hope to increase our understanding of system-wide dopaminergic pharmacology, as well as to provide directions on how to improve pharmacological biomarker strategies during early drug development.

## Methods

A systematic overview of literature over the past 25 years has been built, focusing on dopaminergic treatment effects on central and peripheral biomolecular pathways in rats. A search of the PubMed database was conducted in September 2017 by using the following key words: *dopamine antagonists, dopamine agonists, biogenic amine, amino acid, hormone, cytokine, lipid, neurotransmitter, cerebrospinal fluid, intracerebral microdialysate, plasma, urine, rat* (see Supplementary Data S1 for the exact search code), yielding to 1058 articles (English only). Only studies describing the effects of dopaminergic agents and elucidating a potential biochemical indicator of drug action in rats were included. *In vitro* studies, experimental studies focusing only on behavioral changes and/or reactions, studies of cognition patterns or event-related potentials, and studies that only included pharmacokinetic information were excluded. Furthermore, studies including functional imaging techniques or electroencephalography, investigating dopamine receptor affinities, functions, and synthesis, exploring the effect of dopaminergic agents in combination with other pharmacological agents, under pathological conditions, after surgical procedures such as adrenalectomy or ovariectomy, with pregnant or lactating animals, and with animals under long-term food restriction were excluded as well. Finally, prolactin, being considered a standard marker of dopaminergic activity with well-explored functions and relationship with dopamine (22–24), has been excluded. After selection, 260 articles were included.

## Dopaminergic Treatment Effects on Endogenous Metabolites Levels in the CNS

**Fig. 2:**
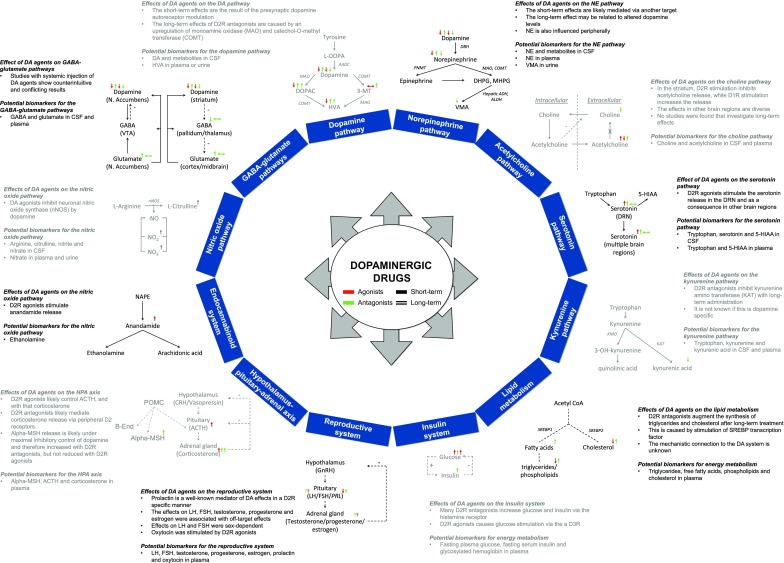
Effects of dopamine drugs on 12 biochemical or endocrine pathways. Potential biomarkers are mentioned for each pathway. The reader is referred to the text for detailed discussion of the interaction between dopamine drugs and each pathway. 5-HIAA: 5-hydroxyindoleacetic acid; ACTH: adenocorticotropic hormone; Alpha-MSH: alpha melanocyte stimulating hormone; B-end: beta-endorphin; COMT: catechol-O-methyl transferase; CSF: cerebrospinal fluid; D1R: dopamine 1-like receptor; D2R: dopamine 2-like receptor; DA: dopamine; DHPG: dihydroxyphenylglycol; DOPAC: 3,4-dihydroxyphenylacetic acid; DRN: dorse raphe nucleus; FSH: follicle stimulating hormone; GABA: gamma-aminobutyric acid; HVA: homovanillic acid; L-DOPA: levodopa; LH: luteinizing hormone; MAO: monoamine oxidase; MHPG: 3-methoxy-4-hydroxyphenylglycol; N. Accumbens: nucleus accumbens; NE: norepinephrine; NO: nitric oxide; NOS: nitric oxide synthase; prolactin: prolactin; VMA: vanillylmandelic acid; VTA: ventral tegmental area.

The CNS-wide effects of dopamine receptor agonists and antagonists reported in the selected studies are shown in Table I and Fig. 2. Although information was also gathered from studies involving intracerebral administration, only data after systemic administration is presented to obtain insights into clinically relevant effects. Moreover, a distinction is made between short-term and long-term treatment effects. Most of the effects reported in the CNS have been mainly observed in brain_ECF_, using microdialysis, leading to deeper insights into neurotransmitter pathways. Overall, the reported literature emphasizes the CNS-wide effects of dopaminergic agents, including dopamine pathway but also norepinephrine, cholinergic, GABA-glutamate, serotonin, kynurenine, nitric oxide and endocannabinoid pathways.

**Table I:**
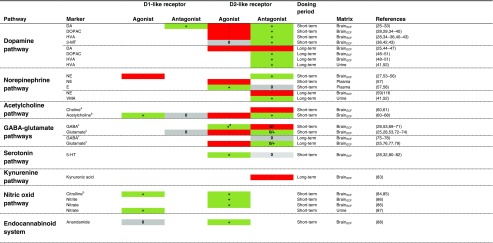
CNS-Wide Effects on Endogenous Metabolites by Dopamine Receptor Agonists and Antagonists

+ (green): increase; - (red): decrease; +/-, -/0 or +/0 (grey): conflicting results; 0 (grey): no effect. In case multiple studies were identified for the effects of a particular drug class on a particular marker, only the 4 most recent publications were reported. ^a^Only in striatum; ^b^Only observations after intracerebral administration; ^c^Few and/or conflicting data; ^d^Measured in the prefrontal cortex

*DA* dopamine, *DOPAC* 3,4-dihydroxyphenylacetic acid, *HVA* homovanillic acid, *3-MT* 3-methoxytyramine, *NE* norepinephrine, *E* epinephrine, *VMA* vanillylmandelic acid, *GABA* gamma-aminobutyric acid, *5-HT* serotonin, *brain*_*ECF*_ brain extracellular fluid

**Fig. 3:**
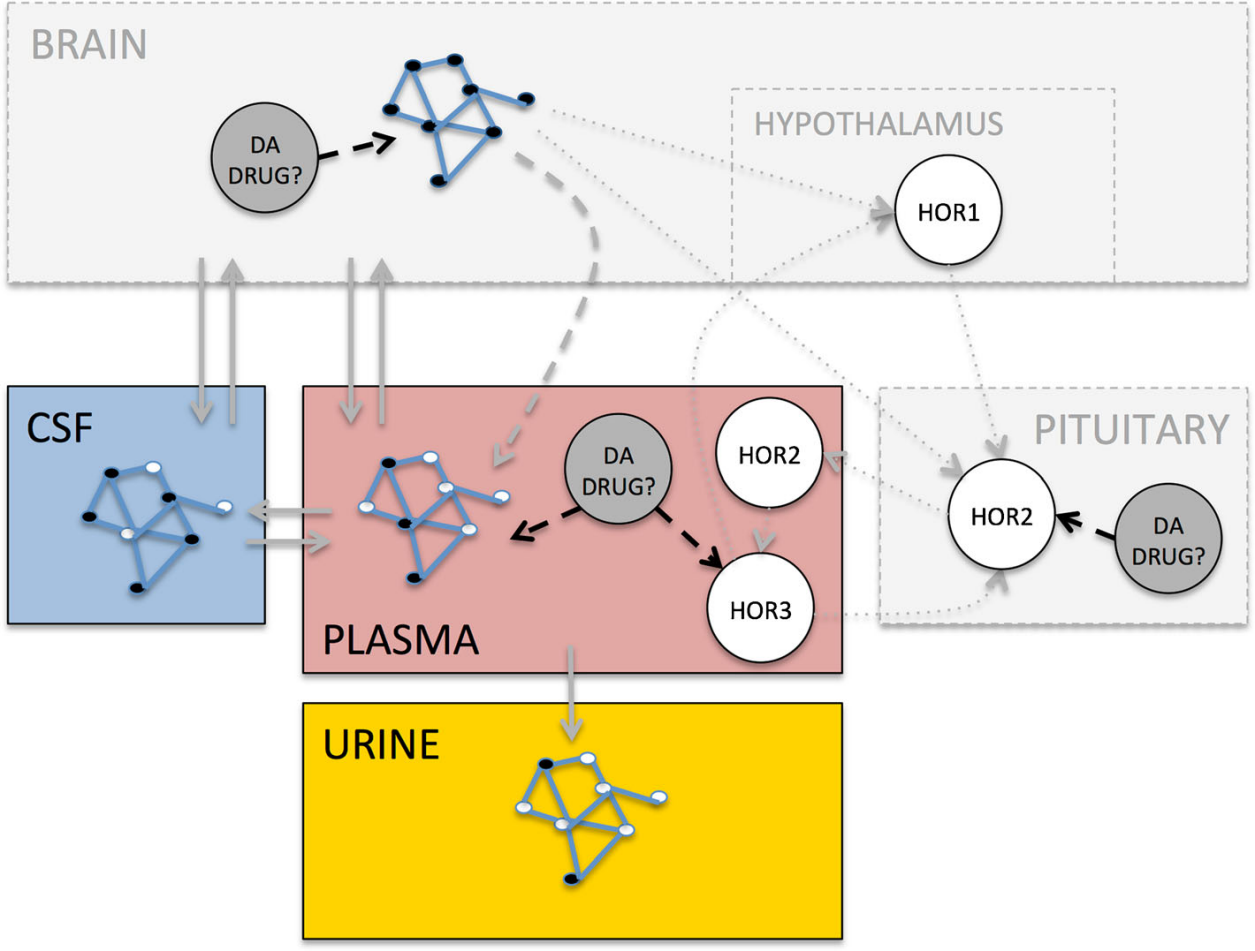
Conceptual considerations for the use of accessible biomarkers in CSF, plasma or urine to reflect dopamine drug effects in the brain. The grey solid lines represent the distribution of biochemical pathway components to CSF, plasma and urine. Since only part of the pathway components may distribute to these biofluids, some of the nodes are filled blank. The grey dashed line represents the peripheral nervous system (PNS) that may influence the peripheral release of biochemical markers through electrical signaling. The grey dotted lines represent the neuroendocrine system (NES), which is electrically controlled at the level of the hypothalamus and the pituitary, causing the release of hormones into plasma. Feedback mechanisms of these hormones on their own release may complicate the interpretation of their responses in plasma. The black dashed lines represent the levels at which dopamine drugs may interact with these systems.

Several considerations have to be taken into account for the discovery of easily accessible biomarkers that reflect these systematic effects, notably (Fig. 3):

i) detectability in CSF, plasma or/and urine;
ii) simultaneous evaluation together with other markers of the pathway of interest to understand the dynamics between the drug and the pathway;
iii) Sufficient understanding of central and peripheral response
iv) Identification of distribution rates between brain, CSF, plasma and urine to understand the temporal relation between the biomarker peripheral concentration and effects in the brain.

### Effects on the Dopamine Pathway

#### Metabolism and Signaling of the Dopamine Pathway

The synthesis of dopamine involves the conversion of tyrosine into L-DOPA, the precursor of dopamine. It is stored into vesicles in the presynaptic neuron, following uptake via the vesicular monoamine transporter (VMAT). These vesicles release dopamine into the synaptic cleft, where it may bind to pre- or postsynaptic dopamine receptors to pass on neuronal signals to the post-synaptic neuron. The dopamine present in the synaptic cleft is eliminated through conversion to its metabolites homovanillic acid (HVA), 3,4-dihydroxyphenylacetic acid (DOPAC) or 3-methoxytyramine (3-MT), or by uptake into the presynaptic neuron via the dopamine transporter. In the latter case, dopamine is stored into vesicles, or degraded to HVA or DOPAC.

#### Effects of Dopaminergic Agents on the Dopamine Pathway

Dopamine receptors are located pre- and postsynaptically, thereby influencing local concentrations of dopamine and its metabolites upon the presence of agonists and antagonists (Table I, Fig. 2). Short-term treatments with D_2_ receptor antagonists such as haloperidol, sulpiride, risperidone, olanzapine and clozapine have shown to stimulate the dopamine pathway (26,28), whereas administration of D_2_ receptor agonists like quinpirole, quinelorane, 7-OH-DPAT, and apomorphine inhibit this pathway (25,30,40). This has been observed in brain_ECF_ for dopamine as well as for its major metabolites DOPAC, HVA, 3-MT (Table I). The influence of D_1_ receptor agents on the dopamine pathway remains poorly investigated. Only one study was identified, showing an increase in dopamine levels after intraperitoneal treatment with the D_1_ receptor antagonist SCH23390 (33), while no studies reported the effects after systemically injected D_1_ receptor agonists. The effects of D_2_ receptor antagonists and agonists on the dopamine pathway may be explained by the modulation of presynaptic D_2_ autoreceptors that provide a negative feedback function on dopamine release (90). Moreover, many of these drugs have affinity for 5-HT receptors (16,21), which also contribute to the control of dopamine release (91,92).

After long-term treatment with D_2_ receptor agonists, the basal dopamine pathway activity is decreased, similar to the effect observed after short-term treatment (25,46). Interestingly, D_2_ receptor antagonists inhibit the dopamine levels after long-term treatment, while the levels of the dopamine metabolites are increased (44,45,93). This may, first of all, be explained by the upregulation of D_2_ receptor expression after long-term treatment (94), thereby leading to an enhanced inhibition of dopamine release via the D_2_ autoreceptor. Second, the monoamine oxidase (MAO) and the catechol-O-methyl transferase (COMT), that metabolize dopamine into DOPAC, HVA and 3-MT, were upregulated (95), providing another explanation, also supporting the increased concentrations of dopamine metabolites that are observed with long-term treatment.

#### Biomarkers for the Dopamine Pathway

Dopamine and its metabolites can be detected in CSF, plasma and urine (52,96). In contrast to dopamine, HVA is able to cross the BBB, providing a way to evaluate central dopaminergic activity in plasma. The difficult aspect is to distinguish between the central and the peripheral effects, since the dopaminergic system is also peripherally active in, for example, the kidney and the adrenal glands. The origin of the HVA response in urine after long-term treatment with haloperidol and clozapine (41,52) is therefore not known. Surprisingly, no further studies were identified that investigated CSF, plasma or urine biomarkers of the dopamine pathway after dopaminergic treatment.

### Effects on the Norepinephrine Pathway

#### Metabolism and Signaling of the Norepinephrine Pathway

The largest concentrations of norepinephrine in the brain are found in neurons in the locus coeruleus. Outside the brain, it is found in the postganglionic sympathetic adrenal fibers and the chromaffin cells in the adrenal glands. Within the norepinephrine neurons, VMAT stores dopamine into synaptic vesicles, where it is converted to norepinephrine through dopamine beta-hydroxylase, and released into the synaptic cleft. Norepinephrine may bind to alpha- or beta-adrenergic receptors, the former being mostly inhibitory and located presynaptically, while the latter are stimulatory and located postsynaptically. From the synaptic cleft, norepinephrine undergoes reuptake into the presynaptic neuron via the norepinephrine transporter, or is metabolized to epinephrine, dihydroxyphenylglycine and methoxyhydroxyphenylglycol. In the presynaptic neuron, it may be stored into vesicles, or degraded into its metabolites.

#### Effects of Dopaminergic Agents on the Norepinephrine Pathway

Norepinephrine release is stimulated by D_2_ receptor antagonists such as clozapine, olanzapine and risperidone, although this has not been reported for haloperidol (27,55) (Table I, Fig. 2). While this may be explained by dopaminergic modulation of norepinephrine release (97), these drugs also exhibit affinity for the adrenergic receptors (16). Interestingly, in contrast to haloperidol, the other D_2_ receptor antagonists showed affinity for the α_2_ adrenergic receptor. After long-term treatment, haloperidol caused a reduction of norepinephrine levels in the striatum (59), which may be explained by reduced conversion from dopamine to norepinephrine, since long-term D_2_ receptor antagonist treatment decreased dopamine levels (Table I, Fig. 2).

Plasma norepinephrine concentrations were decreased after D_2_ receptor stimulation with the agonist bromocriptine (57). This effect was blocked by administration of the D_2_ receptor antagonist domperidone, which does not cross the BBB, suggesting the effect to be peripheral (98). Furthermore, plasma levels of epinephrine were increased upon stimulation of D_2_ receptor, although likely elicited through direct peripheral action on the adrenal gland and independent of the effect on norepinephrine (57,58).

#### Biomarkers for the Norepinephrine Pathway

Norepinephrine and its metabolites have been already analyzed in CSF, plasma and urine (52,57,96), indicating that the latter biofluids can be used to estimate the central norepinephrine pathway activity. Indeed, reduced levels of the most downstream norepinephrine metabolite vanillylmandelic acid were found in urine after long-term treatment with haloperidol or clozapine (41,52). However, as discussed in the previous paragraph, the effect on plasma (and thus also urine) norepinephrine concentrations are at least partly caused by peripheral effects. Further understanding of the relative central and peripheral effects of dopaminergic agents on the plasma or urine norepinephrine pathway responses is needed to conclude whether they can be used as biomarker for central activity. The CSF levels are likely more representative; however, the evaluation of longitudinal norepinephrine pathway responses upon dopaminergic treatment is still lacking.

### Effects on the Acetylcholine Pathway

#### Metabolism and Signaling of the Acetylcholine Pathway

Acetylcholine (ACh) is produced from choline in the presynaptic neurons and stored into vesicles via the vesicular acetylcholine transporter. These vesicles release ACh into the synaptic cleft where it binds to the postsynaptic ACh receptors, which are subclassified into nicotinic receptors that modulate neuronal activity and muscarinic receptors that elicit G-protein dependent signaling. ACh is degraded to choline and acetate, the former being recycled into the presynaptic neuron by the sodium-dependent choline transporter. Interestingly, anticholinergic drugs are typically prescribed to decrease the EPS accompanying antipsychotic treatments, suggesting that the dopaminergic and the cholinergic system are tightly connected. Cholinergic interneurons in the striatum represent only 1–2% of all neurons, yet they play an important role in the integration of multiple neurotransmitter signals (99), thereby contributing to the stabilization of dopaminergic signaling in the psychomotor circuit (also cortico-basal ganglionic system) (100).

#### Effects of Dopaminergic Agents on the Acetylcholine Pathway

As listed in Table I and Fig. 2, ACh release from cholinergic interneurons in the striatum is inversely related to D_2_ receptor stimulation or inhibition. On the other hand, choline, the precursor of ACh, was reduced after D_2_ receptor antagonist treatment, probably as a consequence of ACh release, since the uptake of choline was increased to support ACh production (62,101).

Contrary to their effect in the striatum, D_2_ receptor agonists increased ACh levels in the hippocampus and the frontal cortex (64,102–104). Furthermore, ACh in the PFC and the hippocampus was increased after treatment with second-generation D_2_ receptor antagonists, which was not the case for first-generation D_2_ receptor antagonists (28,103,105–108). ACh levels in the NAc were not affected by D_2_ receptor antagonism (28). Overall, this indicates that the relation between the dopaminergic system and cholinergic signaling is region-specific. Indeed, there is evidence for D_2_ receptor specific regulation of ACh in the striatum, while for other regions the results are conflicting. D_1_ and D_2_ receptors are certainly involved, taking into account that several of the D_2_ receptor binding drugs discussed here also exhibit affinity for the muscarinic receptors (16,103,106).

D_1_ receptor agonists have consistently been reported to lead to increased ACh levels in several brain regions, including the striatum (64,66,68,109,110), while D_1_ receptor antagonism led to decreased ACh concentrations (110), or had no effect (64,103). Cholinergic neurons indeed express the D_1_, mostly the D_5_ receptor, increasing excitability after receptor stimulation (99).

#### Biomarkers for the Acetylcholine Pathway

Both ACh and choline can be detected in CSF and plasma with state-of-the-art analytical methods (111–114). Furthermore, the plasma levels of these molecules may reflect central cholinergic activity, since they both can cross the BBB (115). However, ACh is an important neurotransmitter of the PNS, sending signals from neural endfeet to muscle cells. This might confound the plasma levels as a marker of central activity. Quantitative understanding of the BBB distribution relative to the PNS response is essential to be able to interpret the plasma levels. Moreover, the relation between dopamine treatment and the cholinergic system appeared brain region specific, which may limit the usefulness of CSF and plasma for cholinergic biomarker detection. No studies have investigated cholinergic CSF and plasma in relation to dopaminergic treatment so far. Therefore, it is not possible to conclude whether it is possible to use these biofluids for biomarker evaluation.

### Effects on the GABA-glutamate Pathways

#### Metabolism and Signaling of the GABA-glutamate Pathways

GABA and glutamate are the main inhibitory and excitatory neurotransmitters, respectively, in the brain. Glutamate is synthesized from glutamine by the enzyme glutaminase and is stored in vesicles in glutamatergic neurons via the action of vesicular glutamate transporters. These vesicles release glutamate into the synaptic cleft where it binds to the glutamate receptors, i.e., metabotropic receptor and ionotropic receptors (NMDA, kainate,and AMPA receptors). From the synaptic cleft, glutamate distributes into glial cells, using the glutamate transporter 1 or the glutamate aspartate transporter, where it is metabolized into glutamine. Glutamine is subsequently released from the glial cells and recycled into glutamatergic neurons. Also in GABAergic neurons, glutamate is produced from glutamine. However, these neurons also contain the enzyme glutamate decarboxylase that converts glutamate into GABA. Vesicular GABA transporters store GABA into vesicles which release it into the synaptic cleft. There, it binds to the GABA receptors to inhibit the activity of the postsynaptic neuron. GABA diffuses to the glial cells via the GABA transporter where it is metabolized to glutamate via the Krebs cycle, and subsequently converted to glutamine. Glutamine is recycled into the presynaptic GABAergic neurons. Although glutamate and GABA have many roles in the brain and are distinct neurotransmitters, we discuss here their interconnection in relation to two dopaminergic pathways: the nigrostriatal pathway and the mesocorticolimbic pathway. These pathways belong to the so-called circuits that connect multiple brain regions by neuronal fibers. Concretely, in the nigrostriatal pathway, activation of the striatal D_1_ receptor leads to release of GABA into the internal globus pallidum (GPi) and the substantia nigra reticula (SNr). This subsequently reduces the release of GABA into the thalamus. Activation of the striatal D_2_ receptor inhibits the release of GABA into the external globus pallidum (GPe), which then stimulates the release of GABA into the subthalamic nucleus and the GPi. This also reduces the release of GABA into the thalamus. As such, these two pathways, also referred to direct and indirect pathway, enhance the thalamic release of glutamate into the PFC. Since cortical glutamatergic neurons project to multiple regions in the midbrain, amongst which the striatum and the substantia nigra, many functionalities are stimulated. In the mesocorticolimbic pathway, activation of D_2_ receptors in the VTA stimulates GABAergic neurons in the NAc. This leads to enhancement of GABA release into the other brain regions such as VTA and ventral pallidum. Additionally, D_2_ receptor activation in the VTA stimulates the release of dopamine into the PFC. This enhances the activity of the pyramidal neurons that release glutamate into other brain regions, including NAc and VTA.

#### Effects of Dopaminergic Agents on the GABA-glutamate Pathways

While these circuits for a large part were unraveled by local injection of dopaminergic, GABAergic and glutamatergic agents (116–118), not many studies have been performed showing the effect of systemically injected dopaminergic agents (Table I, Fig. 2). Only one D_1_ receptor agent, an antagonist, was systemically injected to show no effect on glutamate levels in the entopeduncular nucleus (EPN) (74). The cortical GABA levels were increased with systemic injection of D_2_ receptor agonists, while glutamate levels in the NAc or EPN were decreased (25,71,74), contrasting the response expected from the above-described circuits. D_2_ receptor antagonists typically did not show an effect on GABA levels in the ST, the GPe, the PFC and the NAc (28,53,70,72), or glutamate levels in the ST, EPN, PFC or NAc (28,53,73–75,77). It should be noted that the results are not always consistent, since some studies with D_2_ receptor antagonists found reduced GABA levels in the GP, NAc or PFC (70,72,119,120), increased GABA concentrations in the GP or the striatum (76,79), or increased glutamate levels in the SN, ST, EPN, PFC, or NAc (73,75,121,122). These contradictions highlight the delicate balance of this circuit, which is affected by multiple factors (e.g., target site exposure, experiment time, off-target effects, etc.) that can cause concentration-, time-, or drug-dependent differences among the studies. Moreover, with systemic injection, these circuits are perturbed at multiple regions, rendering its pharmacological interpretation non-intuitive. Systematic studies that account for these factors, and that evaluate glutamate, GABA and dopamine in multiple brain regions simultaneously, are warranted to obtain a deeper insight into the effects of systemic administration of dopaminergic agents on such circuits.

#### Biomarkers for the GABA-glutamate Pathways

Although GABA and glutamate concentrations are well measurable with modern analytical approaches (123), it is not known how the levels relate to dopaminergic treatment. GABA and glutamate responses have shown to be region-dependent, which may confound the CSF and plasma response. Further experimental evidence needs to be collected to evaluate the potential of CSF and plasma to assess the GABA-glutamate pathway activity in relation to dopaminergic agents.

### Effects on the Serotonin Pathway

#### Metabolism and Signaling of the Serotonin Pathway

Serotonin is produced from the amino acid tryptophan via 5-hydroxytryptophan and stored into vesicles by VMAT. When it is released from these vesicles into the synaptic cleft, it binds to different classes of 5-HT receptors (5-HT_1_–5-HT_7_). It is recycled into the presynaptic neuron by the serotonin transporter, where it is stored into vesicles or metabolized to 5-hydroxyindoleacetic acid (5-HIAA).

#### Effects of Dopaminergic Agents on the Serotonin Pathway

In contrast, the modulation of serotonin circuits by dopamine is mainly restricted to D_2_ receptor mediated stimulation of serotonin neuron cell bodies in the dorsal raphe nucleus (DRN) that control motor activity. This leads to increased serotonin release in the DRN and other regions such as the striatum (91), as identified with systemic administration of D_2_ receptor agonists (32,81) (Table I, Fig. 2). No effects of dopamine agonists were found on the levels of the metabolite 5-HIAA (35,124). Additionally, it was suggested that D_2_ receptor agonists modulate serotonin afferents presynaptically in the hippocampus (125) or the SN (126). D_2_ receptor antagonists did not show an effect on serotonin levels (28,82,83), except for atypical antipsychotics such as risperidone and clozapine, likely elicited through presynaptic serotonin receptors (16,20,82,83,127). Moreover, 5-HIAA was found increased after risperidone in but not all studies (39,120,128–131).

#### Biomarkers for the Serotonin Pathway

The serotonin metabolite 5-HIAA, but not serotonin itself, has been already detected in CSF (96). serotonin, 5-HIAA and the precursor tryptophan can be also detected in plasma. Although serotonin cannot pass the BBB, the central serotonin pathway activity may be inferred from the tryptophan and 5-HIAA responses. It is, however, important to realize that the serotonin pathway is also present in peripheral systems, for example in platelets. Moreover, tryptophan is provided via food intake. These factors may confound the plasma biomarker response to reflect central activity. Experimental evidence is further needed to investigate the relation between dopaminergic treatments, central serotonin activity and CSF or plasma biomarker responses.

### Interactions Among Neurotransmitter Systems

**Fig. 4:**
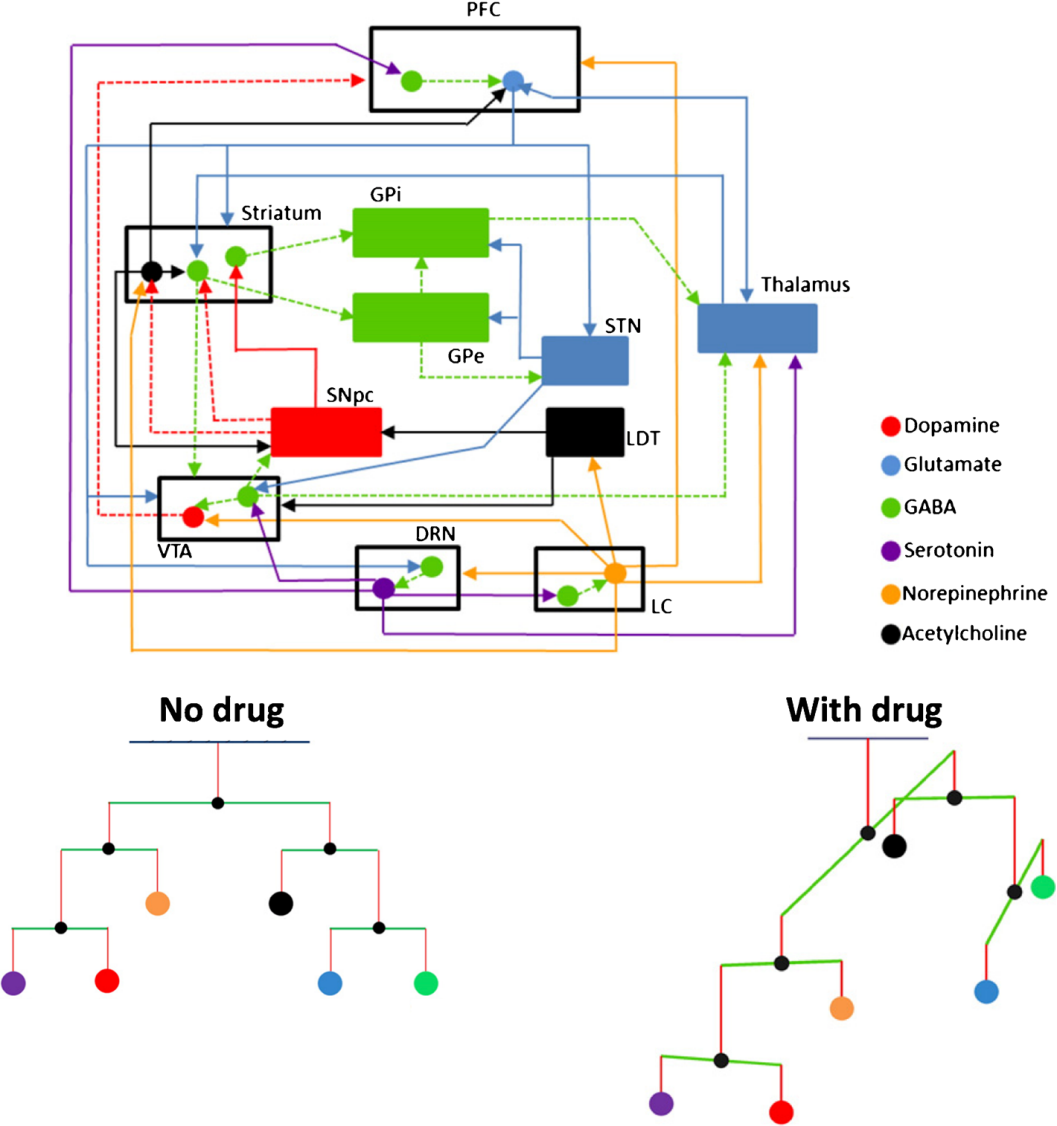
Mathematical model containing expressions for the interactions between the different neurotransmitter systems in multiple brain regions. Rather than looking at single biomarkers, this model enables the prediction of disbalances among the neurotransmitter systems under conditions of drug administration. Adapted from reference (132) with permission.

The above-described effects of dopaminergic agents clearly show that the neurotransmitter systems of dopamine, norepinephrine, GABA, serotonin, glutamate and ACh are highly interconnected. Moreover, many of these agents also influence these neurotransmitter systems via binding to other receptors, such as serotonineric and adrenergic receptors. Therefore, in order to understand the effects of these agents, neurotransmitter responses should be evaluated altogether. Qi et al. (2016) established a network of the connections between these neurotransmitters, taking into account the spatial and functional organization of their neurons and interactions (132) (Fig. 4). This network was used to understand the neurotransmitter disbalances in schizophrenia and their normalization upon antipsychotic treatment. Indeed, disease pathology and drug action must understood in terms of a disbalance among multiple signaling pathways, rather than describing pathology and pharmacology as a single pathway disruption.

#### Biomarkers that Reflect the Balance Among the Neurotransmitter Systems

It will become important to identify accessible biomarkers in CSF, plasma or urine that can reflect the balance among the neurotransmitter systems. While such approach has been followed for a glutamate receptor agonist, identifying the turnover of the dopamine, norepinephrine and serotonin pathway in CSF (96), there has not been such attempt for dopaminergic agents.

### Effects on the Kynurenine Pathway

#### Metabolism and Signaling of the Kynurenine Pathway

Similar to serotonin, kynurenine is a metabolite of tryptophan. In fact, about 95% of tryptophan in the brain is metabolized via the kynurenine pathway, further leading to kynurenic acid, quinolinic acid and 3-OH-kynurenine (133,134). Whereas quinolinic acid is a pro-glutamatergic molecule, kynurenic acid has several anti-glutamatergic properties, such as the antagonism of the NMDA receptor and the inhibition of glutamate release through ACh receptors. 3-OH-kynurenine is involved in the generation of free radicals, independent of the glutamate system (133). 3-OH-kynurenine and quinolinic acid have neurotoxic properties, while kynurenic acid has proven to be neuroprotective (135). A disbalance in the kynurenine metabolism was therefore associated with several neurological disorders, amongst which Parkinson’s disease and schizophrenia (133,136,137).

#### Effects of Dopaminergic Agents on the Kynurenine Pathway

Kynurenic acid was reduced after long-term (1–12 months), but not after shorter-term (1 week) administration of clozapine, raclopride and haloperidol (84) (Table I, Fig. 2). D_2_ receptor antagonists may potentially interfere with the kynurenine amino transferase (KAT) enzyme, which converts kynurenine to kynurenic acid. Indeed, kynurenine and its metabolites other than kynurenic acid were not altered after treatment with D_2_ receptor antagonists (84). It is likely that this effect is D_2_ receptor specific, given that raclopride is a highly selective D_2_ receptor antagonist (138). D_2_ receptor antagonists thus likely inhibit the neuroprotective branch of the kynurenine metabolism, which could be a potential unwanted effect in the long term.

#### Biomarkers for the Kynurenine Pathway

Kynurenine and kynurenic acid are present in sufficient concentration in CSF to be quantified (136,137). Moreover, 40% of the kynurenine synthesis occurs in the brain, while 60% takes place in the blood and is transported over the BBB. It is thus likely that kynurenine and kynurenic acid in CSF and plasma reflect the levels in the brain; however, it is not known to which extent. CSF and plasma levels changes upon dopaminergic treatment remain to be investigated.

### Effects on the Nitric Oxide Pathway

#### Metabolism and Signaling of the Nitric Oxide Pathway

Nitric oxide is generated by nitric oxide synthase (NOS) through the conversion of arginine to citrulline. Nitric oxide has a short half-life (i.e., few seconds) and is readily oxidized to nitrite and nitrate, which can then be measured as an indication of NOS activity. By binding to soluble guanylyl cyclase, nitric oxide stimulates local postsynaptic excitability via modulation of voltage-gated ion channels and possibly also presynaptic neurotransmitter release, thereby modulating synaptic plasticity (139,140). Nitric oxide is tightly connected to glutamatergic signaling. Moreover, it contributes to gonadotrophin and oxytocin release, circadian and respiratory rhythms, locomotor and thalamocortical oscillation, as well as learning process and memory (139). The nitric oxide pathway is downregulated in Parkinson’s disease and schizophrenia, indicating a connection with dopamine (139,141,142).

#### Effects of Dopaminergic Agents on the Nitric Oxide Pathway

Citrulline, nitrite and nitrate have shown to be upregulated after short-term treatment with D_1_ receptor and D_2_ receptor agonists (Table I, Fig. 2). Only two studies with systemic administration have been reported (87,88), while other studies focused on the effects after intracerebral injections (85,86). A possible hypothesis for this upregulation is the stimulation of NOS activity by dopamine, thereby augmenting the production of citrulline and nitric oxide (85). The effect on the nitric oxide pathway was proven to be D_2_ receptor-specific in the striatum (86), while the D_1_ receptor was involved in the NAc (85). Although D_2_ receptor antagonists blocked the effect of D_2_ receptor agonists on nitric oxide concentrations (143), they did not exhibit a significant effect when administered alone (86,144). However, long-term treatment with haloperidol led to an upregulation of neuronal NOS in the hypothalamus (94).

#### Biomarkers for the Nitric Oxide Pathway

Nitrite and nitrate have been measured in the CSF of patients suffering from neurological disorders (141,142), indicating their potential as easily-accessible biomarkers. Nitrate urine levels were found increased after intravenous administration of fenoldopam, a D_1_ receptor agonist, although this effect might have been exerted via D_1_ receptors present in the kidney, rendering difficult to discriminate between peripheral and central effects (88).

### Effects on the Endocannabinoid System

#### Metabolism and Signaling of the Endocannabinoid System

The most well-known components of the endocannabinoid system are anandamide, which is synthesized from N-arachidonoyl phosphatidylethanolamine, and 2-arachidonyl glycerol (2-AG), that is produced from phosphatidylinositol (145). Anandamide is degraded to ethanolamine and arachidonic acid by fatty acid amide hydrolase, while 2-AG is broken down to arachidonic acid by monoglyceride lipase (145). Arachidonic acid is the precursor of a wide range of biologically and clinically important eicosanoids and respective metabolites, including prostaglandins and leukotrienes. The endocannabinoid system is widely distributed in the CNS where it reduces synaptic input through retrograde signaling via cannabinoid receptors, in the brain mainly the CB_1_ receptor subclass (145).

#### Effects of Dopaminergic Agents on the Endocannabinoid System

Dopamine influences the endocannabinoid system mainly in the nigrostriatal pathway by upregulation of endocannabinoid system in the striatum and downregulation in the GPe in a D_2_ receptor dependent manner (146). Indeed, quinpirole stimulated the release of anandamide in the striatum (89), an effect that was blocked by raclopride (Table I, Fig. 2). This provides evidence for D_2_ receptor-dependent involvement of the dopaminergic system in endocannabinoid signaling. Furthermore, although the D_1_ receptor agonist SKF38393 did not cause an effect on anandamide (89), it was found that, with impaired dopamine release, the striatal D_1_ receptor may also affect the endocannabinoid system (146).

#### Biomarkers for the Endocannabinoid System

Even though anandamide can be detected and quantified in the brain, its levels in CSF and plasma are very low (147), rendering its quantitation challenging. Moreover, 2-AG is chemically unstable in aqueous solution, leading to the formation of its isomer 1-AG. Nevertheless, ethanolamine levels can be measured in CSF suggesting this compound as a potential biomarker candidate to reflect the activity of the endocannabinoid system (148).

## Dopaminergic Treatment Effects on the Neuroendocrine and the Energy Systems

Additional to its role in the CNS, the dopamine system is widely expressed in peripheral tissues (10), supporting the importance of evaluating the peripheral effects of dopaminergic agents. The CNS is connected to the periphery via the PNS and the neuroendocrine system, allowing for the opportunity to capture the consequence of central drug effects in the periphery, as done for instance with prolactin (23,24). A significant influence on the hypothalamic-pituitary-adrenal (HPA) axis, the reproductive system, insulin signaling and the lipid metabolism has been found in this systematic review (Table II, Fig. 2). With regards to biomarker discovery, two important aspects can be highlighted (Fig. 3):

i) Biomarkers need to be evaluated together with other markers of the pathway of interest to understand its interaction with the drug;
ii) The connection between brain and target pathway must be quantitatively understood to allow for estimation on how the biomarker response reflects the central effect.

**Table II:**
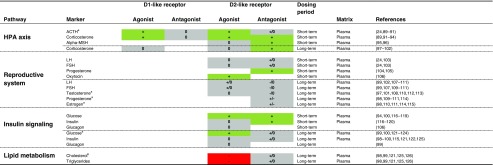
Effects of Dopamine Receptor Agonists and Antagonists on the Neuroendocrine and Energy System

+ (green): increase; - (red): decrease; +/-, -/0 or +/0 (grey): conflicting results; 0 (grey): no effect. + (green): increase; - (red): decrease; +/-, -/0 or +/0 (grey): conflicting results; 0 (grey): no effect.In case multiple studies were identified for the effects of a particular drug class on a particular marker, only the 4 most recent publications were reported. ^a^Few and/or conflicting data; ^b^The atypical antipsychotics risperidone and clozapine showed a positive effect, whereas haloperidol showed a negative effect

*ACTH* adenocorticotropic hormone, *Alpha-MSH* alpha-melanocyte stimulating hormone, *LH* luteinizing hormone, *FSH* follicle stimulating hormone

### Effects on the Hypothalamic-Pituitary-Adrenal (HPA) Axis

#### Signaling in the HPA Axis

The hypothalamo-pituitary-adrenal (HPA) axis is involved in the homeostasis of metabolic and cardiovascular systems, stress response, reproductive system, as well as immune system. It is a complex system of signals and feedback mechanisms between the hypothalamus, the pituitary gland and the adrenal glands. The hypothalamus releases corticotrophin releasing hormone (CRH) and vasopressin to modulate the secretion of adenocorticotropin hormone (ACTH) by the pituitary gland. ACTH subsequently stimulates the release of glucocorticoids (corticosterone in rodents, cortisol in humans) and catecholamines, which control CRH and ACTH release via a negative feedback loop. ACTH is cleaved from the prohormone pro-opiomelanocortin, which also yields to a number of different peptides including alpha-melanocyte stimulating hormone (α-MSH), beta-endorphin and a few other peptides that are also secreted from the pituitary gland.

#### Effects of Dopaminergic Agents on the HPA Axis

A wide range of neural systems influence the HPA axis (185), including dopaminergic system, both in a D_1_ and D_2_ receptor dependent manner (Table II, Fig. 2) (150,151,186). This effect is mainly observed after short-term treatment with D_1_ and D_2_ receptor agonists, while long-term treatment did not show a significant effect on basal ACTH levels (161).

Surprisingly, in contrast to haloperidol, the D_2_ receptor antagonists eticlopride and remoxipride have been reported to increase ACTH plasma levels (24,149). However, remoxipride was 40 times less potent to elicit the ACTH response than to induce the prolactin response (24), suggesting that these observations are explained by off-target effects.

Contrary to their conflicting results for ACTH release, D_2_ receptor antagonists showed a consistent stimulation of corticosterone plasma levels (Table II, Fig. 2), indicating that glucocorticoid release is not only mediated via a central mechanism of ACTH secretion. Additionally, the stimulation of the PNS was suggested to control the sensitivity of the adrenal medulla to ACTH, thereby enhancing the release of corticosterone. It is not certain whether this process is under dopaminergic control, but catecholamines certainly play a role (187). Furthermore, D_2_ receptor antagonists might directly modulate the release of corticosterone, given that D_2_ receptors have been found on the adrenal cortex (188). It is worth mentioning that investigations on dopaminergic innervation in the glucocorticoid release focused on aldosterone release from the zona glomerula, and not on corticosterone release from the zona fasciculate and reticularis (188). Whether the effects of dopaminergic drugs are primarily mediated via dopamine receptors is not fully elucidated. While the ACTH response to D_2_ agonist quinpirole was blocked by the D_2_ antagonist sulpiride, indicating the involvement of the D_2_ receptor, the corticosterone response was not evaluated by such approach (151).

In addition to ACTH and corticosterone, α-MSH secretion from the intermediate lobe of the pituitary gland is also controlled by the dopaminergic system (189). α-MSH levels were increased after D_2_ receptor antagonist treatment (155,156) but changed not after D_2_ receptor agonist treatment (155), suggesting that α-MSH release is under maximal inhibitory control of dopamine.

#### Biomarkers of the HPA Axis

Although the basal mechanisms of the HPA axis are very well understood, it remains unclear at which levels dopamine drugs interfere. The dopamine system is active in the hypothalamus, the pituitary gland, as well as the adrenal gland. While α-MSH and ACTH reflect the response in the pituitary gland upon hypothalamic stimuli, the corticosterone response is secondary to ACTH, or elicited at the adrenal gland directly. Therefore, the interpretation of biomarker responses should rely simultaneous evaluation of α-MSH, ACTH and corticosterone in a longitudinal manner to enable the evaluation of dopamine drug effects at the different levels of the HPA axis.

### Effects on the Reproductive System

#### Signaling in the Reproductive System

The reproductive system also involves communication between the brain and the periphery. It is controlled by the neuroendocrine system through the release of gonadotropin releasing hormone (GnRH) from the hypothalamus, which stimulates the secretion of luteinizing hormone (LH) and follicle stimulating hormone (FSH) in the pituitary gland. These hormones subsequently modulate the release of progesterone and estrogens (estrone, estradiol and estriol) in females, as well as testosterone in males from the reproductive glands, which act as a negative feedback on GnRH release.

#### Effects of Dopaminergic Agents on the Reproductive System

The role of the dopaminergic system in the reproductive system is supported by a well-known side effect of D_2_ receptor antagonists, i.e., sexual dysfunction (190,191). Furthermore, dopamine release in the nigrostriatal, mesolimbic and medial preoptic area plays a crucial role in mating behavior and copulation (192,193), providing a mechanistic basis for the involvement of dopamine in sexual function. Other studies have investigated the dopaminergic drug effects on the sex hormones testosterone, progesterone and estrogen in plasma (Table II, Fig. 2). prolactin was excluded from our analysis because of its well-known relation with dopaminergic agents; however, it is an important mediator of sexual function, supported by the higher frequencies of sexual disorders observed with strong inducers of prolactin (classical antipsychotics and risperidone) compared to weak inducers (e.g., clozapine and olanzapine) (191). The antipsychotic drug-induced disorders are at least partially mediated via peripheral mechanisms, since the peripherally acting D_2_ receptor antagonist domperidone also caused significant changes in reproductive hormones (194).

The results observed for testosterone plasma concentrations were conflicting and mainly associated with high dose levels (157,160,167). Furthermore, while the D_2_ receptor antagonists chlorpromazine and metoclopramide caused a reduction in progesterone and estrogen levels (169,170,173), sulpiride, clozapine, risperidone, and haloperidol led to enhanced concentrations (158,163,164,168). Similarly, LH and FSH were reduced after long-term chlorpromazine and fluphenazine treatment (166,170), while there was no effect observed after long-term sulpiride, risperidone and haloperidol treatment (167,168). After short-term haloperidol treatment, however, increased levels of LH and FSH were observed (162). Interestingly, the effect of short-term D_2_ receptor antagonist treatment was observed in female but not in male rats (24,162).

The non-selective characteristics of the abovementioned D_2_ receptor antagonists may explain these conflicting results, particularly since the effects were associated with large dose levels (16,20). Moreover, sex hormones show a high degree of intra-individual variability and impact of treatment duration, the latter being illustrated by the increased testosterone levels observed after 5 days of domperidone treatment, while it was reduced after 30 days (194). This dual effect highlights the importance of longitudinal sampling upon dopaminergic treatment.

Finally, in addition to the effects of dopaminergic drugs on prolactin and the sex hormones, D_2_ receptor agonists enhanced oxytocin secretion, likely in a D3R-specific manner (165).

#### Biomarkers of the Reproductive System

The reproductive system has multiple levels, i.e., the hypothalamus, the pituitary and the endocrine glands, where further understanding is required to develop an effective biomarker strategy. The prolactin response is already difficult to interpret. Although some studies indicated that it correlates to drug exposure in the brain (23,195), another study found plasma exposure a better predictor (196). A prolactin response has been also observed with domperidone, which does not cross the BBB (194). These observations suggest that the prolactin response is a composite of central and peripheral effects. Similarly, it is not known to which extent LH and FSH represent a central or a peripheral effect. Oxytocin, however, represents a biomarker for central effects only, given that the release is solely controlled by the hypothalamus. The testosterone and progesterone responses are secondary to LH and FSH responses, although they may also have been elicited through a peripheral mechanism. Overall, similar to the HPA axis, the longitudinal evaluation of such possible biomarkers is essential to understand the interaction between dopamine drugs and the reproductive system.

### Effects on the Insulin System

#### Signaling in the Insulin System

It is well known that many antipsychotics, especially atypical, increase the risks for complicated disorders such as metabolic syndrome and type 2 diabetes mellitus (197). Blood glucose levels are controlled by mainly two hormones; insulin and glucagon. Upon a rise in glucose levels, insulin is secreted from pancreatic β-cells, leading to the glucose uptake in the muscles and storage as glycogen in the liver. As a consequence, the insulin secretion is reduced. When blood glucose levels fall, glucagon is released from the pancreatic α-cells, causing glucose release from the liver.

#### Effects of Dopaminergic Agents on the Insulin System

Although insulin signaling is under PNS control (198), the role of dopamine is mainly at the periphery. It is argued that dopamine and insulin are co-secreted from the pancreatic beta cells, with dopamine providing a negative feedback on insulin secretion in a D_2_-like receptor dependent manner (199). However, both insulin and glucagon levels were not influence by short-term D_2_ receptor agonist treatment (Table II, Fig. 2) (177), highlighting that this mechanism does not play a major role. In contrast, glucose concentrations were increased after treatment with the D_3_ agonist 7-OH-DPAT, which was antagonized by raclopride. Interestingly, this effect was confirmed for quinpirole, but not for bromocriptine (177). Possibly, off-target mechanisms of bromocriptine normalize the D_3_ receptor mediated effect on glucose. Both glucose and insulin levels were increased with D_2_ receptor antagonists (Table II, Fig. 2). Typically, the dose required to elicit a short-term glucose response was higher than the one needed for a corticosterone response (154), indicating that an off-target effect explains this response.

The results of long-term treatment are conflicting, with in general no effect on basal fasting glucose or insulin levels (93,158,160,179), although for some D2 receptor antagonists a stimulation of the insulin system has been observed (93,160,180,183). Given the large variation in experimental design (sex, strain, fasting protocol, dose levels), it is difficult to identify the source of this discrepancy. Moreover, many D_2_ receptor antagonists were found to share the off-target affinity for other receptors, such as serotonine, muscarinic and the histamine receptor, all involved in weight gain which is associated with insulin resistance and hyperglycemia (16,197,200). Interestingly, the M_3_ muscarinic receptor was found to be crucial in the control of insulin release (201). It is thus likely that the short- and the long-term effects of D_2_ receptor antagonists on the insulin system are mediated via other receptors than the D_2_ receptor only.

#### Biomarkers of the Insulin System

The insulin system has been well described in terms of biomarkers, including fasting plasma glucose, fasting serum insulin and glycated hemoglobin. Systematic and well-controlled studies that longitudinally evaluate these biomarkers in combination with dopamine treatment are needed to better understand their potential interaction.

### Effects on the Lipid Metabolism

#### Metabolism and Signaling in the Lipid System

Phospholipid and cholesterol pathways are the main pathways of lipid metabolism. Both pathways start with acetyl CoA, and depending on whether the enzyme SREB-1 or SREB-2 is present, the fatty acid or the cholesterol pathway is activated (158). Fatty acids are subsequently converted to triglycerides or phospholipids, amongst others. Cholesterol and phospholipids are notably essential to maintain the cell membrane integrity (202). A distorted lipid metabolism can lead to the loss of neural transmission and is involved in brain several disorders, including schizophrenia (203). Moreover, misbalances in the lipid homeostasis may, for example, cause weight gain, atherosclerosis and cardiovascular problems. In this regard, the relation between dopaminergic drugs and the lipid metabolism is closely related to what is observed with the insulin system (197,204).

#### Effects of Dopaminergic Agents on the Lipid System

The lipid metabolism has shown to be significantly altered after long-term treatment, while no studies were identified for short-term treatment (Table II, Fig. 2). For instance, 2–3 week treatment with the D_2_ receptor antagonists risperidone and olanzapine caused an increase in triacylglycerols and a decrease in free fatty acids plasma levels, which was not the case for the partial D_2_ receptor agonist aripiprazole (18). Another study showed that 4 weeks of treatment with clozapine and risperidone, but not haloperidol, raised the serum levels of total cholesterol, free fatty acids and triglycerides via modulation of the pathway that is responsible for their biosynthesis (158). The fact that the D_2_ receptor agonist ergocryptine, although relatively unselective for this receptor, has been reported to decrease total cholesterol and triglycerides concentrations (159), may indicate that these effects are mediated via D_2_ receptors. However, given that not all D_2_ receptor antagonists affect the lipid metabolism, other receptors than the D_2_ receptor may be involved. Further investigations are needed to investigate through which mechanism dopaminergic agents affect the lipid metabolism.

#### Biomarkers of the Lipid Metabolism

Cholesterol, free fatty acids, triacylglycerols and triglycerides can be used as biomarker to evaluate dopamine treatment effect on the lipid metabolism. Additionally, a lipidomics-based approach also revealed an increase of phosphatidylethanolamine as biomarker for antipsychotic efficacy (18).

## Recommendations for Biochemical Biomarker Strategies in CNS Drug Development

This review provides an extensive overview into the effects of dopaminergic agents on multiple biological pathways in the CNS and the periphery, as well as the potential of easily accessible biomarkers to reflect these effects. Overall, there is a strong need for systematic searches for biomarkers that together can represent the system-wide effects of dopaminergic agents. Here, we provide the following recommendations to account for system-wide effects in early CNS drug development.

### Use Proteomics and Metabolomics-Based Biomarkers Discovery for CNS Drug Effects

We envision a crucial role for proteomics and metabolomics approach to further elucidate known and unknown pathways and to identify drug effect-related biomarkers (205). Considering the potential lack of insights into the system-wide effects of a new compound in early drug development, these methodologies enable preclinical anticipation of wanted and unwanted effects (206). This information can then be used to optimize the future dosing strategies. Also, using a targeted metabolomics approach with monoamines in the brain, it was shown that risperidone and clozapine are biochemically closer to the 5-HT_2A_ antagonist M100907 than to haloperidol (116,207). Interestingly, this pattern highly corresponded with behavioral outcome (116). Indeed, many of the dopaminergic agents described in this review are non-selective. Pharmacological effects should be seen as a balance between multiple components of a network of affected biochemical pathways (Fig. 4) (132). CNS drug discovery should thus aim for rational development of non-selective drugs to attack the polygenic CNS disorders (16). Proteomics and metabolomics will certainly provide additional and valuable tools for the investigation of the *in vivo* pharmacology (205).

### Use Longitudinal Data and Mathematical Modeling

Mathematical modeling to understand CNS drug effects are further needed. A pharmacological interaction at one or more receptors will pass on to the neurotransmitter network, causing the net result on the individual neurotransmitters, as well as the balance between them, being not so intuitive. A mathematical evaluation is therefore needed to understand CNS drug effects (132,208,209). In this regard, longitudinal data on biomarker levels is essential to calibrate these models. Indeed, the pattern of the response reveals information that cannot be obtained from single time point measures (4,210). For example, it was observed that not only basal levels of dopamine and norepinephrine were decreased after long-term treatment, but also the effect size after pharmacological stimulation (44,59). Moreover, it is also difficult to quantify the effect by a single time point in short-term treatments.

### Evaluate CNS Drug Effects in Combination with Pharmacokinetics

This temporal pattern not only depends on the dynamic interactions within the biological system, but also on the exposure pattern of the drug and its possible active drug metabolites at the site of action. It is therefore important to take into account the pharmacokinetics when evaluating the pharmacodynamics. Only one study considered the steady state plasma concentrations of clozapine and its active metabolite N-desmethylclozapine in combination with a response measure [75]. The levels of the drug and the metabolite showed high variability between the animals. Moreover, the ratio between clozapine and its metabolite was dependent on the sex of the animal and the dose. Given the fact that the exposure of the drug and its metabolite drives the response, such variability can have a significant impact on the biomarker plasma levels. This is particularly true for CNS drugs, for which the exposure pattern in the brain is determined by a complex interaction of pharmacokinetics, BBB transport and distribution through the brain (209). Moreover, the drug exposure is likely to be brain region-specific, which will lead to quantitative differences in drug-receptor interactions, depending on the brain region (211). Thus, when pharmacokinetics is taken into account, pharmacodynamics can be compared between drugs of the same pharmacological class, excluding the interference of pharmacokinetic differences.

### Analyze Brain, Plasma and CSF Biomarkers Simultaneously

Plasma (or urine) samples are typically used for biomarker identification, while CSF samples are getting more and more interest in CNS-related diseases. Interestingly, our literature search did not reveal pharmacological biomarker evaluations in CSF, even though it has been used for other drug classes (96) and discovery of pathological biomarkers (123). Although plasma and CSF have the advantage to be accessible in humans, biomarker responses in these biofluids may give a biased view with regard to the actual effects in the brain. Many biomarkers, for example dopamine, do not cross the BBB. Even in the case they do (e.g., HVA) or if the biomarker is measured in CSF, it is difficult to know how is quantitatively relates to the effects in the brain. The current overview shows hardly any studies that simultaneously studied biomarker responses in brain_ECF_ and plasma. One study measured plasma and brain cholesterol levels after long-term treatment with clozapine or haloperidol, but no significant correlation was found (212). Another study could positively associate serum progesterone levels with brain allopregnanolone as a reflection of GABA_A_ potentiation and anxiolytic effect after short-term treatment with olanzapine and clozapine (163). Systematic and simultaneous biomarker evaluations in plasma and brain are recommended to provide a quantitative relation between the central effect and the accessible biomarker response.

### Investigate the Condition-Dependency of Pharmacological Effects

Dopaminergic effects are highly condition dependent. As an illustration, dopamine receptors are present on immune cells to reduce their activation level (213,214), but no effect of dopaminergic agents was found on immune markers such as C-reactive protein, interleukin-6 or tumor-necrosis-factor alpha (160,182,215,216). On the other hand, haloperidol was found to have immune-modulatory and anti-inflammatory effects in an animal disease model of rheumatoid arthritis (217). Indeed, D_2_ receptor antagonists have been shown to normalize lipopolysaccharide-induced inflammation (218), indicating that only in an activated immune system, D_2_ receptor antagonists have an effect on immune markers. Thus, while some markers may not respond under healthy conditions, these observations cannot directly be extrapolated to a diseased condition. Patients or diseased animals need to be evaluated as a population on its own.

## Conclusions

This review highlights that dopaminergic agents, even selective ones, have a wide array of biochemical effects. Indeed, dopaminergic drugs may interfere with at least 8 different systems in the brain, including dopamine signaling, norepinephrine signaling, ACh signaling, GABA-glutamate circuits, serotonin signaling, kynurenine metabolism, nitric oxide pathway, endocannabinoid system, and 4 systems in the periphery, i.e., HPA axis, reproductive system, insulin signaling, and lipid metabolism. Moreover, in line with earlier reviews, many dopaminergic drugs are non-selective (16,20,21). Therefore, although we refer to ‘dopaminergic drugs’, the biochemical actions of these drugs may be elicited via non-dopamine receptors. A systems pharmacology approach is expected to provide deeper insight into the actions of dopaminergic drugs. With such approach it will become possible to anticipate unwanted effects, such as weight gain or sexual disorders. It is stressed that CNS drug development lacks accessible biomarkers that represent central effect. Hardly any studies were found that relate the central effect to an accessible (i.e. CSF, plasma, urine) biomarker response. Moreover, plasma samples were mostly obtained at a single time-point, thereby missing the insight into the longitudinal pattern of the effect. Overall, given that other neurotransmitter systems are similarly interconnected as the dopamine system and also widely expressed, we highlight the need for longitudinal system-wide biomarker evaluations to create greater understanding of CNS and to improve early CNS drug development.

## ABBREVIATIONS

3-MT: 3-methoxytyramine;
5-HT: Serotonin
5-HIAA: 5-hydroxyindoleacetic acid
α-MSH: Alpha-melanocyte stimulating hormone
ACh: Acetylcholine
ACTH: Adenocorticotropic hormone
BBB: Blood-brain-barrier
Brain_ECF_: Brain extracellular fluid
CNS: Central nervous system
CRH: Corticotropin releasing hormone
CSF: Cerebrospinal fluid
DOPAC: 3,4-dihydroxyphenylacetic acid
DRN: Dorse raphe nucleus
EPN: Entopeduncular nucleus
EPS: Extrapyramidal symptom
FSH: Follicle stimulating hormone
GABA: Gamma-aminobutyric acid
GnRH: Gonadotropin releasing hormone
GPe: External globus pallidum
GPi: Internal globus pallidum
HPA: Hypothalamic-pituitary-axis
HVA: Homovanillic acid
LH: Luteinizing hormone
MSN: Medium spiny neuron
NAc: Nucleus accumbens
NMDA: N-methyl-D-aspartate
NOS: Nitric oxide synthase
PFC: Prefrontal cortex
PNS: Peripheral nervous system
PVN: Paraventricular nucleus
SN: Substantia nigra
VMAT: Vesicular monoamine transporter
VTA: Ventral tegmental area
